# Momentary emotion-related distress, negative emotions, and suicidal ideation: Findings from an RCT among adults in inpatient psychiatric care

**DOI:** 10.1016/j.jad.2026.121628

**Published:** 2026-03-13

**Authors:** Annabelle M. Mournet, Kate H. Bentley, Hannah R. Krall, Matthew Flics, Mariah T. Hawes-Sousa, Elizabeth A. Edershile, Christopher AhnAllen, Evan M. Kleiman

**Affiliations:** a Rutgers, The State University of New Jersey, New Brunswick, United States; b Massachusetts General Hospital, Boston, United States; c Harvard Medical School, Boston, United States; d Rutgers University Behavioral Healthcare, Piscataway, United States; e Brigham and Women's Hospital, Boston, United States

**Keywords:** Suicidal ideation, Emotion-related distress, Sadness, Negative affect, Psychiatric inpatients

## Abstract

**Objective:**

Examine the moderating role of distress about emotions (i.e., emotion-related distress) in the relationship between specific negative emotions and suicidal ideation (SI) among adults hospitalized for psychiatric reasons in an RCT of a brief cognitive-behavioral therapy (CBT)-based intervention. Specifically, we investigate how randomization to the study intervention versus control condition impacts these relationships.

**Method:**

Participants (*N* = 64) were recruited from psychiatric inpatient units at two hospitals as part of a larger RCT. Participants in the treatment group completed three 1:1 structured therapy sessions focused on CBT skills for managing emotions and a 28-day ecological momentary assessment/intervention (EMA/EMI) protocol facilitating skills practice after discharge. Participants in the control condition completed post-discharge EMA only. Both groups received treatment as usual. Multi-level modeling examined the relationships between each emotion assessed via EMA (i.e., anxious, sad, overall negative emotion) with SI, moderated by emotion-related distress and study condition.

**Results:**

The three-way interactions between study condition, emotion-related distress, and emotion predicting contemporaneous SI were significant (anxiety: *b* = 0.05 [95% CI = 0.02–0.09], *p* = .006; sadness: *b* = 0.05 [95% CI = 0.02–0.09], *p* = .006; negative affect: *b* = 0.05 [95% *CI* = 0.02–0.09], *p* = .006). Among intervention condition participants, emotion-related distress was a significant moderator between each emotion and SI, with stronger associations at higher levels of emotion-related distress, compared to average and low levels of emotion-related distress.

**Discussion:**

Emotion-related distress moderated associations between negative emotions and SI in an intervention-specific manner, suggesting CBT-based EMI may target proximal risk processes for SI among individuals with high emotion-related distress.

## 1. Introduction

Adults in inpatient psychiatric care are a group with historically high rates of distress about emotions and suicidal ideation (SI; Steer et al., 1993). Post-inpatient discharge is also a high-risk time for suicide, given the transition from a high-support to low-support environment, high rates of ailed transition to lower levels of care, and difficulty continuing to use therapy skills outside of the inpatient unit (Forte et al., 2019). Accordingly, interventions are needed to support individuals in inpatient psychiatric care post-discharge, particularly with continuing to use evidence-based coping skills learned during their inpatient stay as well as to reduce suicide risk and to address other distressing emotions.

Extensive research has highlighted the association between emotion dysregulation, or the inability to effectively manage one's emotions, with suicidal thoughts and behaviors (STBs; Turton et al., 2021). A recent systematic review provided an in-depth examination of the many studies related to emotion regulation and STBs but highlights that the majority were cross-sectional and that there is a dearth of longitudinal research on this topic (Turton et al., 2021). Related to emotion dysregulation is distress about emotions (i.e., emotion-related distress), defined as physical or emotional discomfort and pain resulting from any negative mood state, including sadness. While this phenomenon and related phenomenona have been described using many different terms, in this manuscript, we refer to “emotion-related distress” to represent the extent to which an individual feels distressed by how they are feeling emotionally. Related (but distinct) terms exist, such as “distress tolerance” that generally refers to the extent to which an individual can withstand distressing emotions (Zvolensky et al., 2010), whereas “emotional distress” more often refers to the unpleasant state of significant mental stress or suffering (Matthews, 2016). Importantly, we view emotion-related distress to be distinct from these other terms in that emotion-related distress does not speak to the extent to which that distress can be tolerated (i.e., distress tolerance), which may vary from person to person, nor does it speak to the general valence of the emotion, which could be pleasant or unpleasant (i.e., in emotional distress the emotion is inherently unpleasant, whereas with emotion-related distress, emotions such as joy may be distressing to individuals who are used to experiencing sadness [e.g., Joshanloo, 2013]).

STBs can happen in relation to uncomfortable negative emotions, such as sadness (Kuehn et al., 2024; Turton et al., 2021) and can serve as a form of negative affective reinforcement (Kleiman et al., 2018). Given the pain and discomfort that emotions can produce, it is valuable to understand how specific emotions relate to STBs. For instance, leveraging the 2019 National Youth Risk Behavior Survey, researchers found experiencing sadness to be associated with 8 to 11 times greater odds of suicidal behavior (Trimble and Chandran, 2021). Additionally, numerous studies have found significant associations between the role of negative affect and STBs using an ecological momentary assessment (EMA) approach (Gee et al., 2020). For instance, in a 4-week EMA study of individuals with first-episode psychosis, within-person emotion regulation interacted with between-person negative affect and predicted concurrent SI (Wastler et al., 2025). In an EMA study among university students, difficulty inhibiting emotional responses to self-harm images did not directly predict stronger NSSI urges, but it did amplify urges during moments of high negative affect (Burke et al., 2021). Similarly, Kuehn et al. (2024) found sadness to be associated with contemporaneous suicidal thoughts in an EMA study. EMA is particularly well-suited for research questions focused on a smaller timescale, given frequent fluctuations in emotional states (Schoevers et al., 2021) and SI (Kleiman et al., 2017). This finely grained data collection method allows for enhanced understanding of constructs that change rapidly in a short period of time.

As research increasingly establishes associations between emotion dysregulation, specific emotional states (e.g., sadness, anger) and STBs (Ammerman et al., 2015; Bentley et al., 2020; Cui et al., 2023; Gee et al., 2020; Kuehn et al., 2024), there is a need to investigate how to leverage this information to reduce suicide risk. The Unified Protocol for Transdiagnostic Treatment of Emotional Disorders (UP; Barlow et al., 2011, 2018) is a cognitive behavioral therapy (CBT) intervention that focuses on delivering adaptive skills for managing emotional states and countering emotion-driven avoidance (Barlow et al., 2020). Traditionally, there are five modules in the UP treatment: mindful emotion awareness, cognitive flexibility, preventing emotional avoidance, tolerating physical sensations related to emotions, and emotion-focused exposures (Barlow et al., 2011). The UP has been found to be efficacious for the treatment of emotional disorders, as evidenced by meta-analytic research (Carlucci et al., 2021) and has been studied in numerous settings, including psychiatric inpatient settings (Bentley et al., 2017). Existing proof of concept and pilot work adapting the UP in inpatient contexts has been found to be feasible and acceptable (Bentley et al., 2017; Bentley et al., 2020).

Given the relationship between emotion dysregulation and STBs (Turton et al., 2021), a randomized controlled trial (RCT) was designed to understand whether an ecological momentary intervention (EMI) can support real-time use of therapy skills for individuals following psychiatric inpatient care to reduce distress (NCT05848089 and NCT06555094). This EMI RCT leveraged three specific CBT emotion management skills from the UP given the efficacy of transdiagnostic CBT skills for adaptive emotion management as an approach to reduce STBs (Ghahramanlou-Holloway et al., 2015; Stanley et al., 2009). These skills are: 1) Anchoring in the Present from the mindful emotion awareness module, 2) Thinking Flexibly from the cognitive flexibility module, and 3) Changing Emotional Behaviors from the preventing emotional avoidance module. EMIs, which typically involve mobile phones, are increasingly popular given their accessibility and scalability (Balaskas et al., 2021). Several existing studies are utilizing an EMI approach to treat suicide risk, however many of these studies are currently ongoing (Barrigon et al., 2022) or have focused exclusively on acceptability and feasibility (Morgiève et al., 2022). Of note, the feasibility and acceptability of EMI to support the usage of CBT emotion management skills among post-discharge adult psychiatric inpatients has already been reported on using the presently analyzed dataset (Kleiman et al., 2024).

The present study utilizes data from the aforementioned recent RCT to examine the role of emotion-related distress in the concurrent relationship between specific negative emotions and SI among adults in inpatient psychiatry care. Specifically, we investigate whether a CBT-based emotion management EMI is effective by reducing emotion-related distress. Emotion-related distress may moderate the relationship between emotional experiences and SI given that the extent to which an emotion causes distress can vary from person to person. One individual may find the emotion of sadness deeply distressing, while others may be able to experience sadness without feeling substantial distress associated with the emotion. With emotion-related distress strongly associated with STBs (Turton et al., 2021), emotion-related distress may serve as an impactful variable on the relationship between specific emotions and SI. Moreover, neurocognitive work suggests that individuals with suicidal ideation demonstrate altered processing of emotional stimuli, including biased attention and neural reactivity to facial affect (Yin et al., 2026), which may contribute to heightened emotional distress and downstream suicide risk. Delivering and practicing skills for adaptive emotion management may decrease levels of SI associated with emotions because individuals may be able to experience unpleasant emotions without intense distress, preventing the onset or worsening of SI. Information on how these variables related to one another will provide valuable insight into the efficacy of this EMI-based intervention and mechanisms of action.

## 2. Method

### 2.1. Participants

Participants for this study were recruited from psychiatric inpatient units at two hospitals, Rutgers University Behavioral Healthcare (UBHC) and Brigham and Women's Faulkner Hospital (BWFH), as part of a larger RCT (Rutgers NCT06555094, BWFH NCT05848089). IRB approval was obtained from both study sites. To participate in the RCT, individuals needed to be 18 years of age or older and admitted for treatment of mood-related problems (e.g., depression, anxiety) and/or STBs. Individuals with active symptoms that prevented the ability to provide informed consent (e.g., interfering psychosis or mania) or current aggressive behavior were excluded from participating.

### 2.2. Procedures

New admissions to the inpatient units were screened by study staff for preliminary eligibility using the electronic medical records. A study team member received permission to approach each potential participant from the clinical staff. Potentially eligible individuals were approached and individuals who were interested completed the informed consent process in a private space. Following consent, participants completed a series of baseline questionnaires. Participants were then randomized to the study intervention or control condition.

Participants in the intervention condition completed three therapy sessions with a study clinician. The three sessions were focused on three skills, each adapted from a distinct UP module. The choice to only include three skills was to ensure the brevity of this intervention, given time restrictions for providing therapy to psychiatric inpatients as well as to try to ensure that skills did not conflict with other interventions received during that time. The UP skills used in this brief psychotherapy intervention were: 1) Anchoring in the Present, 2) Thinking Flexibly, and 3) Changing Emotional Behaviors. Anchoring in the Present helps individuals focus on the present moment, rather than the past or future. Thinking Flexibly guides individuals through a series of questions designed to help them consider other potential interpretations of situations to reduce rigid thinking. Finally, Changing Emotional Behaviors involves considering alternative adaptive coping strategies to use in place of maladaptive behaviors that occur in response to strong emotions. During each session, the therapist taught the skill and then practiced the skill with the participant. The therapy sessions occurred predominantly while patients were on the unit, with any remaining sessions completed remotely via Zoom/phone as soon as possible after discharge, if the participant was discharged before receiving all sessions. All participants also received all usual inpatient and post-discharge care.

**Fig. 1.**
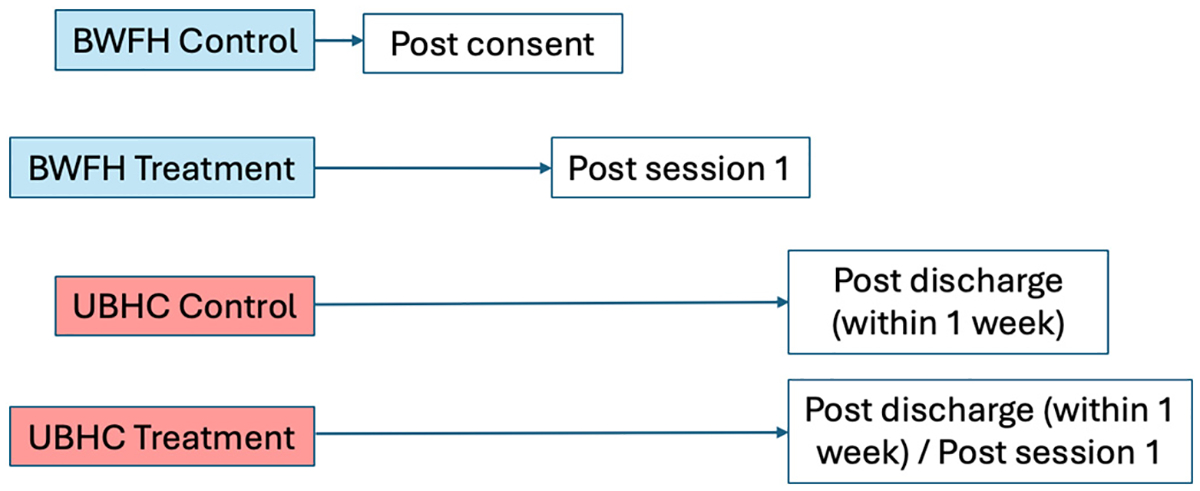
MetricWire enrollment process based on condition and study site.

Participants in the control condition completed survey items (i.e., EMA only), whereas the intervention group participants completed survey items and were prompted to practice the therapy skills, equating to EMI. EMA/EMI were completed via MetricWire. MetricWire enrollment varied based on treatment versus control group as well as study site (see Fig. 1). Treatment group participants were enrolled in MetricWire only after they had completed at least one of the therapy skills. At BWFH, patients are typically permitted to keep their smartphone with them during their hospitalization. Thus, BWFH control group participants began EMA on the unit, immediately after completing the consent/baseline visit. BWFH treatment group participants began EMI after the first therapy skill was completed. Patients at Rutgers UBHC are generally not allowed to have their smartphones on the unit, so participants from this site began EMA within one week of being discharged from the hospital. Research assistants met with participants on the unit (BWFH) or over the phone/zoom (UBHC) to guide participants through downloading and logging into the app. EMA/EMI consisted of a 28-day protocol. All participants received four random surveys, with at least 2 h between surveys, as well as one evening survey (at a fixed time) per day. Participants set their own waking hours during which to receive random surveys and the time of their evening survey to increase compliance. Surveys for both groups included items related to current affect, SI, and for the treatment group, skills practice.

### 2.3. Measures

#### 2.3.1. Baseline

Participants completed a battery of self-report questionnaires at baseline, including a demographics questionnaire as well as the SelfInjurious Thoughts and Behaviors Interview—Self Report (SITBI-SR; Nock et al., 2007). The SITBI-SR is a self-report questionnaire used to assess SI, as well as other facets of self-injurious thoughts and behaviors. For the current analyses, several SITBI-SR items were used to characterize the sample. Participants also completed baseline measures of depression and anxiety, specifically the Patient-Reported Outcomes Measurement Information System (PROMIS) Anxiety 4a – Short Form (PROMIS, 2023a) and the PROMIS Depression 4a – Short Form (PROMIS, 2023b), to characterize the psychopathology of the sample. Raw scores were converted to normed T-scores (M = 50, SD = 10).

#### 2.3.2. Emotional states

Emotion states were assessed through EMA using a set of single items adjectives. The adjectives used in the current study were “anxious”, “sad”, “and “negative” (“negative” is a proxy for overall negative affect). All emotional state items were asked in the form of: “Right now, how [emotional state] do you feel?” All emotional states were rated on a an 11-point scale from 0 (Not at all) to 10 (Very much).

#### 2.3.3. Emotion-related distress

One EMA item was used to measure emotion-related distress: “Right now, I am distressed by how I'm feeling (emotionally).” Participants responded to this item on an 11-point scale from 0 (Not at all) to 10 (Very much).

#### 2.3.4. Suicidal ideation

Three EMA items were used to assess SI. These three items have been used in prior EMA work (Glenn et al., 2022; Kleiman et al., 2018): “Right now, how strongly do you wish you weren't alive anymore?”; “Right now, how strongly do you want to kill yourself?”; “Right now, how strong is your intent to kill yourself?” Each item was rated on an 11-point scale from 0 (Not at all) to 10 (Very much). At each observation, a composite score was created to represent overall SI.

### 2.4. Data analysis

Data were analyzed using R, version 4.3.2. Participants with at least two EMA surveys were included in analyses. A series of Fisher's exact tests and *t*-tests were used to examine demographic and clinical differences between individuals included in the analyses and those excluded due to missing data. Using the R package, *lme4* (Bates et al., 2015), linear mixed effect multi-level models were performed examining the relationship between each emotion state (anxiety, sadness, and negative affect) with the composite SI score. A multilevel confirmatory factor analysis was used to estimate within- and between-person components of the latent construct of SI and to confirm that that creating a composite SI variable is appropriate. The multilevel CFA demonstrated a within-person alpha of 0.70 and a between-person alpha of 0.86, supporting the aggregation of these related but non-identical SI variables into a composite construct. Each emotion predictor as well as emotion-related distress was person-mean centered. Three-way interactions were included in each model, to examine the moderator of emotion-related distress as well as the impact of study condition. Models with significant interactions were probed using the Johnson-Neyman probe. All models were also run controlling for baseline depression as well as SI at the previous momentary assessment. Given differences in study site, including different EMA starting points, a sensitivity analysis was performed for each of the three models to control for study site.

## 3. Results

Across both sites, a total of 104 individuals consented to participate in the study (including the EMA portion), were enrolled in the study, and were randomized. Of these individuals, forty completed zero or one EMA surveys (38.5%), and due to a large amount of missing data, were not included in analyses. 61.5% of participants had 2 or more EMA surveys, resulting in a final sample of 64 participants who completed the EMA portion of the study and are included in the present analyses. The EMA compliance rate was 40.6%. Thirty-eight of these participants (59.4%) participated at BWFH, with the remaining 26 participants at Rutgers UBHC (40.6%). Participants had a mean age of 32.7 years (SD = 12.0). The sample included 43.8% men, 42.2% women, and 14.1% another gender identity. Regarding race and ethnicity, 59.4% of participants identified as White, 14.1% Black, 6.3% more than one race, 20.3% another racial identity, and 25.0% Hispanic. Thirty-four participants (53.1%) were randomized to the treatment group, whereas 30 participants (46.9%) were randomized to the control group. There were no significant differences in age, gender, race, nor ethnicity by study condition (i.e. treatment vs. control). The majority of participants (90.6%; 58/64) reported lifetime SI and 80.0% of participants reported past week SI (51/64). There were no significant differences in STBs by study condition nor between the two study sites. At baseline, participants had a mean anxiety score of 66.89 (SD = 7.12) and a mean depression score of 66.17 (6.34). There were no significant differences age, gender, ethnicity, SI, nor anxiety between participants who were included in the analyses compared to those excluded due to missing data. Participants excluded from the analyses due to missing data were more likely to be Black (*p* = .03) and had lower levels of depression reported at baseline (*p* = .02). Participant characteristics are summarized in Table 1. Table 2 provides descriptive statistics of SI based on RCT condition.

### 3.1. Anxiety

**Fig. 2.**
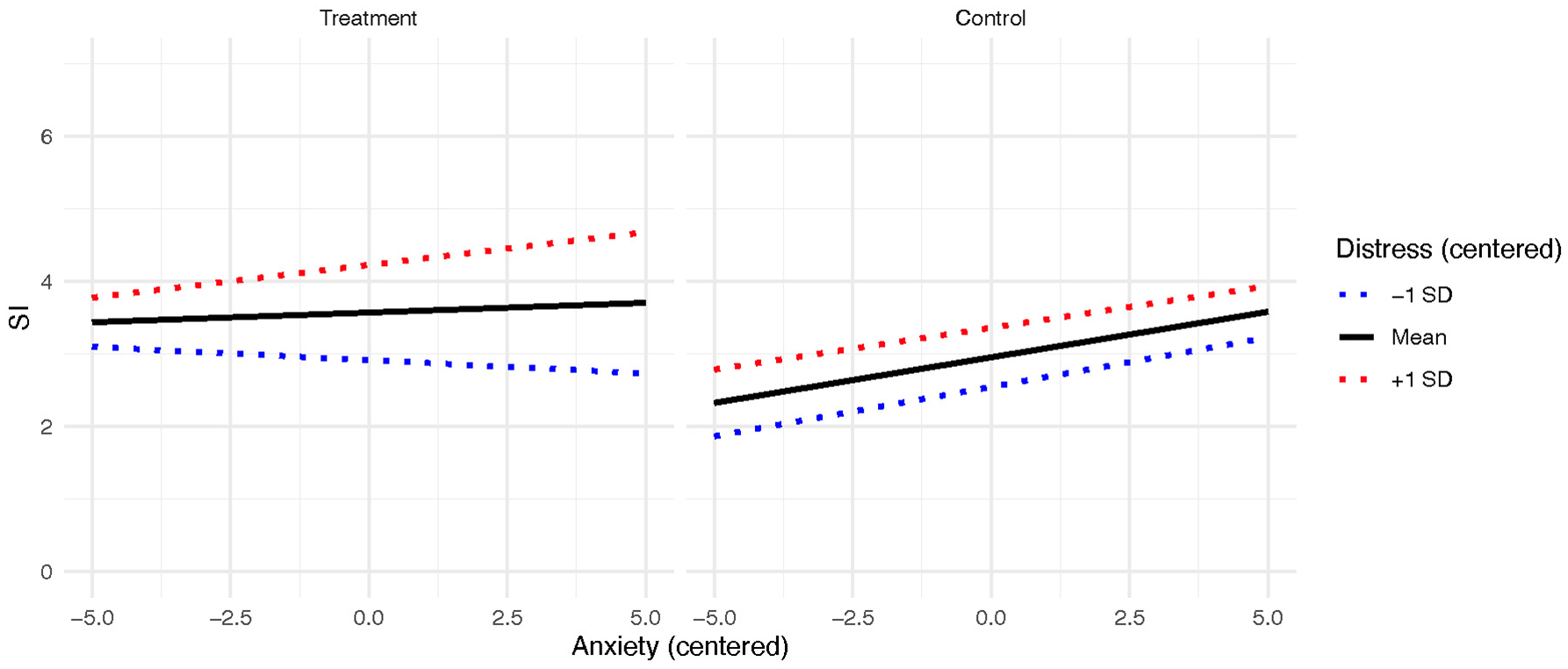
Anxiety and SI relationship moderated by emotion-related distress and RCT condition.

For the anxiety model, the main effects for anxiety (*b* = 0.13 [95% CI = 0.04–0.21], *p* = .003) and emotion-related distress (*b* = 0.41 [95% CI = 0.33–0.48], *p* < .001) on SI were significant. When examining the two-way interactions, the emotion-related distress X study condition interaction was significant (*b* = 0.25 [95% CI = 0.14–0.36], *p* < .001). The three-way interaction between study condition, emotion-related distress, and anxiety predicting SI was also significant (b = 0.05 [95% CI = 0.02–0.09], *p* = .006). This three-way interaction is plotted in Fig. 2. When examining the simple slopes in the treatment group, emotion-related distress significantly moderated the relationship between anxiety and SI such that the relationship between anxiety and SI was significant at high (+1 SD) levels of emotion-related distress (b = 0.14, *t* = 2.98, *p* < .001) but not average (b = 0.03, *t* = 0.58, *p* = .56) and low (−1 SD; b = −0.09, *t* = −1.66, *p* = .10) levels. When examining the simple slopes in the control group, there was no significant difference between levels of emotion-related distress (b range from 0.11 to 0.15, t ranges from 6.65 to 7.99, p ranges from <0.001–0.03).

### 3.2. Sadness

**Fig. 3.**
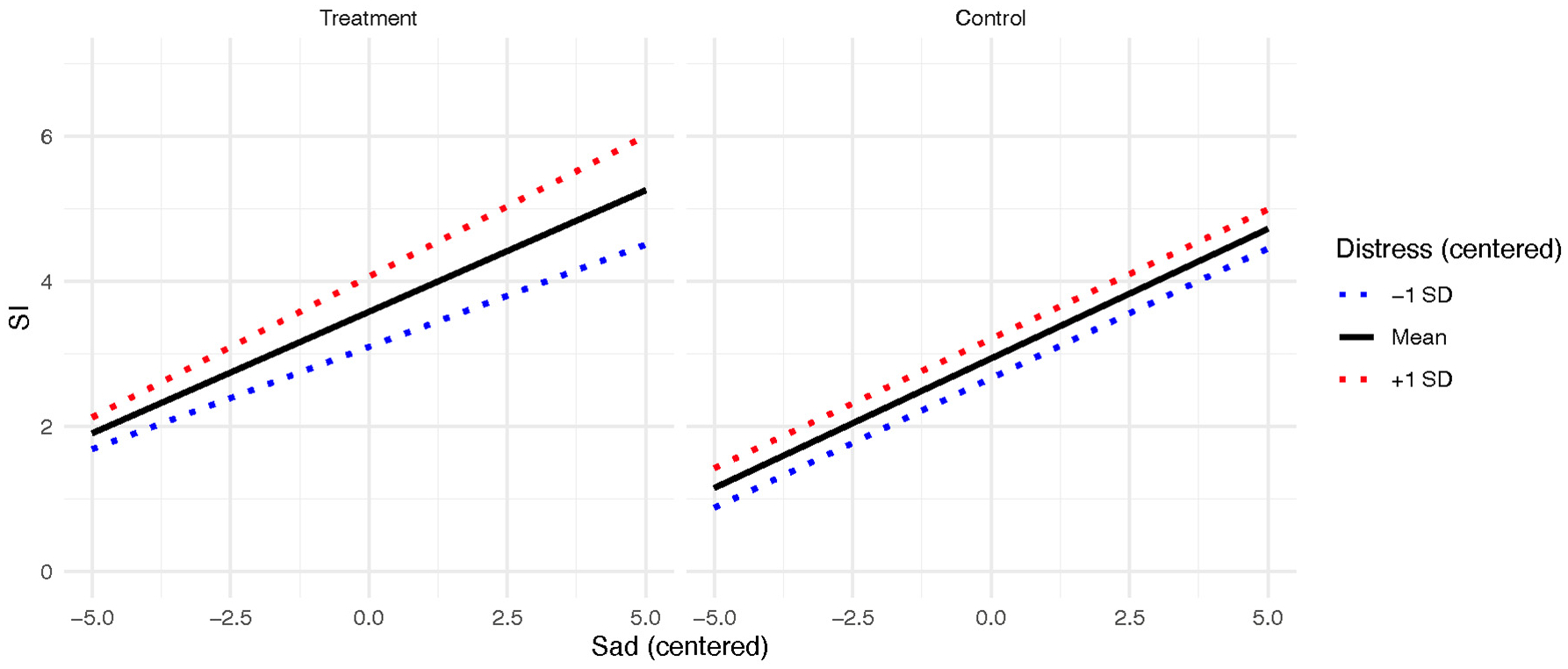
Sadness and SI relationship moderated by emotion-related distress and RCT condition.

For the sadness model, the main effects for sadness (*b* = 0.36 [95% CI = 0.27–0.44], *p* < .001) and emotion-related distress (*b* = 0.27 [95% CI = 0.19–0.35], *p* < .001) on SI were significant. For the two-way interactions, the emotion-related distress X study condition interaction was again significant (b = 0.21 [95% CI = 0.10–0.32], *p* < .001). The three-way interaction between study condition, emotion-related distress, and sadness predicting SI was also significant (b = 0.05 [95% CI = 0.02–0.09], *p* = .006). This interaction is plotted in Fig. 3. When examining the simple slopes probes in the treatment group, emotion-related distress significantly moderated the relationship between sadness and SI such that the relationship between sadness and SI was stronger at high (+1 SD) levels of emotion-related distress (b = 0.43, *t* = 9.26, *p* < .001) compared to average (b = 0.33, *t* = 7.85, *p* < .001) and low (−1 SD; b = 0.24, *t* = 4.46, *p* < .001) levels. When examining the simple slopes in the control group, there was no difference between levels of emotion-related distress (b = 0.36 for high, low, and average, t ranges from 6.65 to 7.99, p < .001).

### 3.3. Negative affect

**Fig. 4.**
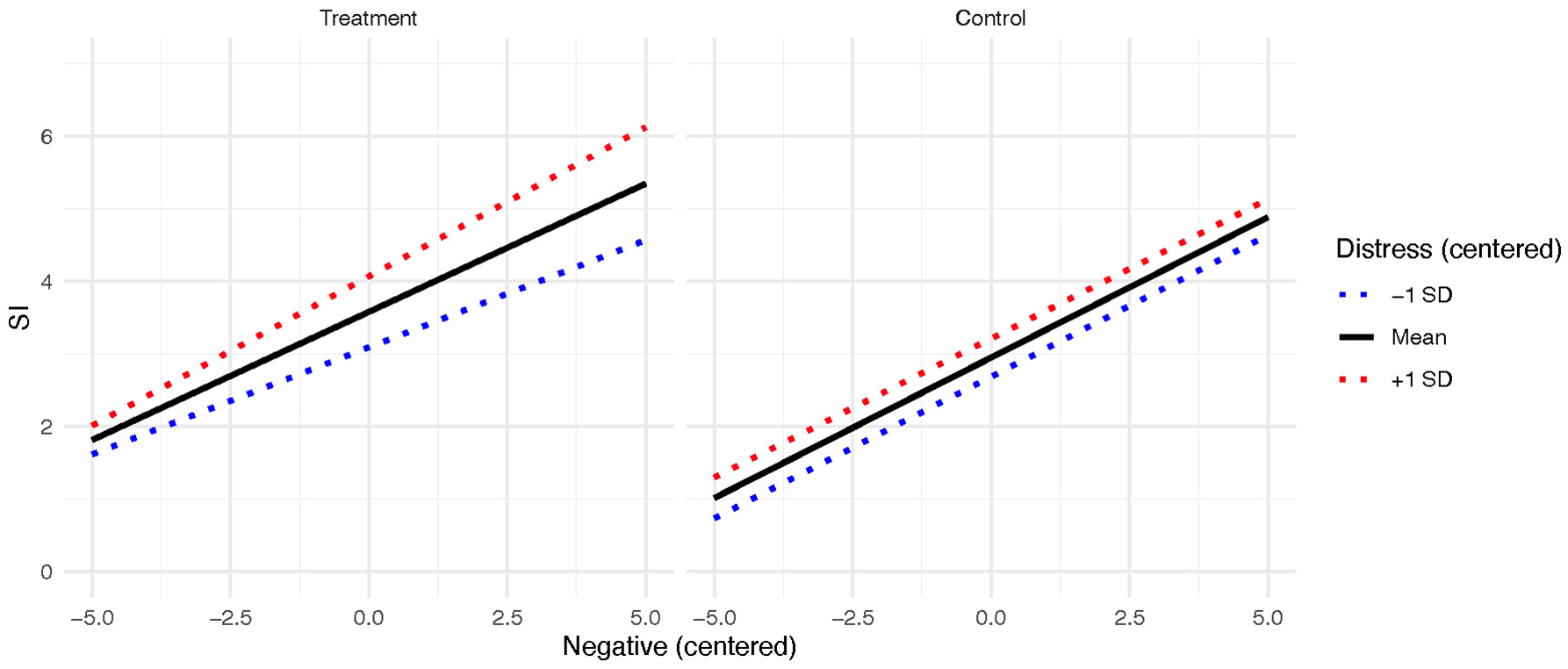
Negative affect and SI relationship moderated by emotion-related distress and RCT condition.

In the negative affect model, which refers to overall negative affect measured with one item, the main effects for negative affect (*b* = 0.39 [95% CI = 0.30–0.47], *p* = .003) and emotion-related distress (*b* = 0.26 [95% CI = 0.18–0.34], *p* < .001) on SI were significant. With regards to two-way interactions, the emotion-related distress X study condition interaction was significant (*b* = 0.22 [95% CI = 0.11–0.34], *p* < .001). The three-way interaction between study condition, emotion-related distress, and negative affect predicting SI was also significant (*b* = 0.05 [95%CI = 0.02–0.09], *p* = .006). This interaction is plotted in Fig. 4. When examining the simple slopes probes in the treatment condition, emotion-related distress significantly moderated the relationship between sadness and SI such that the relationship between sadness and SI was stronger at high (+1 SD) levels of emotion-related distress (*b* = 0.46, *t* = 9.43, *p* < .001) compared to average (*b* = 0.35, *t* = 8.27, *p* < .001) and low (−1 SD; *b* = 0.24, *t* = 4.79, *p* < .001) levels. When examining the simple slopes in the control group, there was not a significant difference between levels of emotion-related distress, although both slopes differed from 0 (b ranges from 0.38 to 0.39, t ranges from 6.65 to 7.99, p < .001).

When controlling for baseline depression and SI at the previous EMA survey, three-way interaction findings were consistent with the original models. A sensitivity analysis also revealed that controlling for site did not substantively impact the main findings.

## 4. Discussion

The primary goal of this investigation was to examine three-way interactions between three emotional states differing in arousal and specificity, emotion-related distress, and study condition on the outcome of SI. Greater levels of each emotion – anxiety, sadness, and negative affect – as well as emotion-related distress, were all associated with greater overall SI. The decision to examine associations with a composite SI variable were supported by multilevel CFA findings of related but non-identical facets of the original three SI items. Findings were largely consistent across each emotion state, wherein for treatment group participants, emotion-related distress was a significant moderator between each emotion state with SI. The relationships between each emotion and SI were stronger at higher levels of emotion-related distress, compared to average and low levels of emotion-related distress. This was not the case, however, in the control condition. In the intervention condition, participants who felt less distressed by emotions, sadness, anxiety, and negative affect did not have as strong of a relationship with SI. Results reveal that for participants who received CBT emotion management skills and help practicing those skills through EMI, when they feel distress from their emotions, these emotions continue to be related to their SI; when participants feel less distress from their emotions the level of emotion is not associated with SI. This may be attributable to CBT skills practice improving how individuals respond to emotion-related distress.

With regards to two-way interaction effects, the only significant interaction effect detected was the emotion-related distress X study condition interaction. This interaction was significant across all three emotion models, demonstrating that study condition significantly moderated the relationship between emotion-related distress and SI. In other words, the study intervention appears to have created a “buffer” that disrupts the link between specific emotions and SI during periods of low and average emotion-related distress. Such findings are to be expected, given that the intervention group received skills specifically targeting emotion management. Results support the efficacy of transdiagnostic emotion management CBT skills to reduce emotion-related distress associated with emotions and accordingly reduce SI. Indeed, prior research has demonstrated that the full UP treatment reduces anxiety and depression by means of improvement in emotion regulation (Khakpoor et al., 2019). The current analyses further extend prior work to show that these emotion management skills may be effective at reducing links between specific negative effects and SI in higher-risk samples and with a briefer course of treatment.

Findings from this secondary analysis of RCT data also revealed that an EMI designed to support the use of emotion management skills in everyday life appears to have a proximal relationship to the association between negative emotions and SI among adults in psychiatric inpatient care. Findings also suggestion that emotion management skills that help individuals feel less distress when they experience a negative emotion may lead to less momentary distress from these emotions and less SI. This appears to be a potentially helpful adjunctive strategy for suicide prevention efforts among a high-risk population.

One somewhat unexpected finding was that when participants experienced high levels of momentary anxiety and low levels of distress, they also were less likely to report SI. Though unexpected, this may be reflective of the Yerkes-Dodson Law wherein certain levels of stress can be associated with improved performance (Yerkes and Dodson, 1908). Applied to the current findings, higher than average anxiety levels, when not associated with emotion-related distress may be motivating to some individuals. Greater levels of intrinsic motivation have been marked as a potential protective factor against STBs (Britton et al., 2014). Accordingly, there may be value to further investigating mediating constructs in the relationship between high anxiety and lower levels of SI. Alternatively, this finding could simply reflect the intervention working effectively; individuals who were feeling anxious but not distressed by anxiety were less likely to report SI.

Valuable clinical implications arise based on the present findings. In this study, the use of EMI to support adults in psychiatric inpatient care in utilizing skills for emotion management revealed significant momentary findings. This intervention involved administering three brief manualized therapy sessions to participants and setting them up with a smartphone app to encourage continued use of these skills beyond the three sessions. Given the feasibility and acceptability of such an approach (Kleiman et al., 2024), the current efficacy for mental health outcomes revealed through the present analyses underscores the value of this approach. This approach aligns with emerging intervention research demonstrating that structured programs targeting emotion regulation and interpersonal skills (e.g., MERITA group therapy) produce meaningful improvements in emotional functioning among high-risk populations, reinforcing the value of scalable, skills-based treatments that directly target emotion-related mechanisms in suicide prevention (Alvarez-Segura et al., 2025). Other psychiatric inpatient units should seek to implement a similar approach given the limited time needed from clinicians and the increasing accessibility and scalability of EMI technology (Balaskas et al., 2021).

There are numerous avenues for future research based on the present findings. Future research on this topic with larger sample sizes would benefit from exploring between-subject differences by including between- and within-subject terms for affect states and emotion-related distress variables, to further clarify the relationship between these variables. Future work should also more directly examine potential reactivity effects of EMA monitoring itself, as repeated assessment of emotions and suicidal ideation may function as a low-intensity intervention by increasing emotional awareness. Research designs that compare solely treatment as usual, EMA-only, EMI-only, and combined approaches will help clarify whether buffering effects reflect unique benefits of CBT-based skills, synergistic effects with monitoring, or both.

Additionally, the focus of the present analyses is on moderation. There would be value to future research utilizing a panel or a mediation model analytic approach to examine how variables such as emotion-related distress precede or follow a particular emotion, and how that relationship impacts SI. Finally, future research should further explore the extent to which SI is caused by emotion related distress in and of itself, regardless of affect state.

### 4.1. Limitations

Although the present study has numerous strengths, including the use of a control condition as well as an intensive data collection design, there are several limitations that should be considered when interpreting findings. For one, based on differing policies about phone access on the inpatient unit, there were differences in start dates of EMA/EMI by site, particularly at Rutgers UBHC where participants may have had a longer period between receiving the therapy sessions and beginning EMA/EMI. Relatedly, there was variation in terms of the amount of time that occurred between each session, which may impact findings. Importantly, these variations were naturalistic to the inpatient units and accordingly are likely to reflect the reality of providing brief manualized therapy coupled with EMI to psychiatric inpatients. Additionally, given the relatively small sample size, the stability of the detected interaction estimates is low. Moreover, the EMA compliance rate was 40.6% which is lower than compliance rates in many community-based EMA studies (e.g., 40–94% [Wang et al., 2025], 23–94% [Williams et al., 2021]), however this rate is comparable to other research among individuals with elevated mental health concerns (e.g., 38% [Armey et al., 2011]; 44% [Coppersmith et al., 2019]) and may reflect the substantial burden and instability experienced during inpatient psychiatric care. Notably, Black participants were significantly more likely to be excluded from analyses due to missing data, highlighting potential disparities in engagement with EMA protocols. These findings underscore the importance of considering racial and socioeconomic factors when designing EMA studies and interpreting compliance rates. In this study, we also measured the construct of emotion-related distress with a single item. While this is a common approach in EMA research, there is a need for further testing of this item to further establish the construct validity of the item. Lastly, while we characterize the anxiety and depression levels of participant at baseline, we lack specific diagnostic information for participants. Future studies should examine differences in the efficacy of brief, scalable interventions based on psychiatric diagnoses.

## 5. Conclusions

With high rates of STBs among adults in psychiatric inpatient care, interventions that are scalable and effective are needed to support this high-risk population. A brief, three session version of the UP may represent a valuable approach to address risk for individuals hospitalized due to mental health challenges. Skills such as Anchoring in the Present (Barlow et al., 2011, 2018) may have the ability to help individuals identify negative emotions and experience less momentary distress from these emotions. This in turn, has the potential to be a valuable strategy to reduce negative emotions and associated SI.

## Figures and Tables

**Table 1:** Study, demographic, and clinical characteristics (*N* = 64).

Study characteristic	N (%)/M (SD)
Site	
BWFH	38 (59.4%)
Rutgers UBHC	26 (40.6%)
Condition	
Treatment	34 (53.1%)
Control	30 (46.9%)
Demographic characteristic	
Age	32.7 (12.0)
Gender	
Men	28 (43.8%)
Women	27 (42.2%)
Another gender identity	9 (14.1%)
Race	
White	38 (59.4%)
Black	9 (14.1%)
Multiracial	4 (6.3%)
Another racial identity	13 (20.3%)
Ethnicity	
Hispanic	16 (25.0%)
Non-Hispanic	48 (75.0%)
Clinical characteristic	
Lifetime suicidal ideation	58 (90.6%)
Past week suicidal ideation	51 (80.0%)
Baseline anxiety	66.89 (7.12)
Baseline depression	66.17 (6.34)

Note: Baseline anxiety and depression scores are normed T-scores (M = 50, SD = 10).

**Table 2:** Descriptive statistics of SI by RCT condition.

Condition	Treatment	Control
	N/%	N/%
Total participants	34	30
SI overall	27	24
SI passive	27	23
SI desire	20	17
SI intent	14	15
	N/%	N/%
Total surveys	2009	1627
SI overall	44.10%	41.20%
SI passive	43.90%	40.40%
SI desire	35.30%	31.60%
SI intent	17.10%	19.60%

Note: Top half of table represents number of participants who endorsed each SI type on at least one EMA survey (indicated by a score of >0). Bottom half of table represents the percentage of all EMA surveys where each SI type was endorsed (indicated by a score of >0).
